# Acute interleukin-10 predicts persistent post-concussion symptoms in children with mild traumatic brain injury: exploratory findings from the t-BIOMAP study

**DOI:** 10.3389/fneur.2026.1893730

**Published:** 2026-08-18

**Authors:** Anne-Cécile Chiollaz, Virginie Pouillard, Michelle Seiler, Céline Habre, Fabrizio Romano, Céline Ritter Schenk, Fabian Spigariol, Christian Korff, Fabienne Maréchal, Verena Wyss, Lyssia Gruaz, Jean-Charles Sanchez, Sergio Manzano

**Affiliations:** 1 Internal Medicine Department, Faculty of Medicine, University of Geneva, Geneva, Switzerland; 2 Pediatric Neurology Unit, Woman, Child and Adolescent Department, Geneva University Hospitals, Geneva, Switzerland; 3 Pediatric Emergency Department, University Children's Hospital Zurich, Zurich, Switzerland; 4 Division of Radiology, University Hospitals of Geneva, Geneva, Switzerland; 5 Division of Pediatric Emergency Medicine, Department of Pediatrics, Inselspital, Bern University Hospital, University of Bern, Bern, Switzerland; 6 Department of Pediatrics, Fribourg Hospital HFR, Fribourg, Switzerland; 7 Pediatric Emergency Department, Neuchâtel Hospital (RHNE), Neuchatel, Switzerland; 8 Platform of Pediatric Clinical Research, Woman, Child and Adolescent Department, Geneva University Hospitals, Geneva, Switzerland; 9 Pediatric Emergency Department, Geneva University Hospitals and Faculty of Medicine, University of Geneva, Geneva, Switzerland

**Keywords:** blood-biomarkers, children, cytokines, emergency department, mTBI, post-concussion symptoms, prognosis

## Abstract

**Background:**

Children are a vulnerable population for mild traumatic brain injury (mTBI), accounting for a high burden of emergency department (ED) visits. A significant and unpredictable subset will develop persistent post-concussion symptoms (P-PCS), with consequences for daily quality of life. At ED discharge, no reliable tool exists to identify children requiring neuropsychological follow-up. Acute blood biomarkers represent a promising approach for early risk stratification.

**Methods:**

In this prospective multicenter cohort study, children with mTBI had a blood sample collected within 24 h of injury for biomarker analysis (S100b, NfL, NTproBNP, GFAP, HFABP, IL6, IL10). The parent version of the Post Concussion Symptom Inventory (PCSI-P) was sent at 14 days and 3 months post-injury. P-PCS were defined as the presence of three or more symptoms worsening by at least one point above pre-injury baseline at both timepoints. This analysis included only children identified to be symptomatic at 14 days. Within this group, biomarker levels were compared between those with P-PCS at 3 months and those who had recovered.

**Results:**

Of 203 eligible children, 120 (60%) completed the 14-day questionnaire and 84 (41%) completed the 3-month questionnaire. Among the 38 children who remained symptomatic at 14 days, 16 (42%) met criteria for P-PCS at 3 months, 9 (24%) recovered, and 13 (34%) were lost to follow-up. IL10 levels were significantly elevated in children with P-PCS (*p* = 0.014), with an AUC of 79.9% (95% CI: 59.5–100%). At the optimal Youden index threshold, IL10 achieved 100% sensitivity and 55.6% specificity for ruling-out P-PCS. At 100% specificity, IL10 achieved a sensitivity of 31% for ruling-in P-PCS.

**Conclusion:**

This exploratory study suggests that early IL10 measured at ED presentation may support two complementary risk-stratification strategies: a rule-out approach (100% negative predictive value [NPV]) to safely exclude children unlikely to develop P-PCS, and a rule-in approach (100% positive predictive value [PPV]) to identify those at high risk of requiring neuropsychological follow-up. Consistent with adult TBI literature, these findings highlight the acute neuroinflammatory response in pediatric recovery. Replication in larger cohorts is needed before clinical application.

## 1 Introduction

Mild traumatic brain injury (mTBI) is one of the most frequent causes of emergency department (ED) visits in children worldwide, representing a major public health concern (1–3). Although the overall prognosis of pediatric mTBI is generally favorable, a clinically significant proportion of children, estimated between 10 and 30%, develop persistent post-concussion symptoms (P-PCS) (4–6). These symptoms constitute a substantial burden for children and their families, impairing school performance, social functioning, and overall quality of life (7).

A fundamental challenge in this context is that P-PCS are inherently non-specific. Symptoms such as headache, fatigue, concentrating difficulties, emotional reliability, and sleep disturbance are common complaints and their attribution to a head injury requires careful clinical judgment (8, 9). Unlike other acquired neurological conditions, no standardized predictive tool exists for mTBI-related PCS in children. The most widely cited clinical prediction model is the Predicting and Preventing Postconcussive Problems in Pediatrics (5P) clinical score, derived by Zemek et al. from a large prospective multicentre cohort of children, which incorporates nine clinical variables (age, sex, prior concussion, headache, noise sensitivity, fatigue, answering questions slowly, and emotional symptoms) and demonstrated an AUC of 0.71 (6). While promising, this model was developed and validated in a North American context, and its external validity across different healthcare systems remains limited. Definitive assessment relies on comprehensive neuropsychological evaluation by trained specialists, a process that is time-consuming, costly, and dependent on access to scarce expertise (10). As a result, children who develop P-PCS are often identified late, when early intervention would likely be most beneficial (6).

Given the high number of pediatric mTBI presentations, indiscriminate follow-up of all patients is neither feasible nor resource efficient. There remains a need for objective tool applicable at ED discharge that may assist in identifying children at increased risk of developing P-PCS. Such an approach would better target referrals while avoiding unnecessary burden on families and healthcare systems.

Blood-based biomarkers have emerged as promising candidates for objective assessment following TBI. Biomarkers such as S100 calcium-binding protein B (S100b) and glial fibrillary acidic protein (GFAP) have primarily been investigated for their ability to detect intracranial injury and to guide computed tomography (CT) decision-making in both adult and pediatric populations (11–15). However, their utility in predicting functional outcomes and symptom trajectory following mTBI remains limited and largely unestablished (16, 17), particularly in children. In adults, inflammatory markers such as interleukin 10 (IL10) and heart fatty-acid binding protein (HFABP), measured within 24 h of injury, have been identified as early predictors of functional outcome assessed by the Glasgow Outcome Scale Extended (GOSE) at 6 months post-injury, outperforming more established markers such as S100b, GFAP, and NfL (18). The neuroinflammatory response triggered by mTBI has increasingly attracted attention as a potential determinant of recovery trajectories. Pro- and anti-inflammatory cytokines are rapidly released following brain trauma and have been associated with clinical outcomes (19, 20). Despite this growing interest, no blood biomarker has yet been validated for routine clinical use to predict the long-term outcome of pediatric mTBI patients.

The present study is part of the t-BIOMAP project, a prospective multicenter cohort of children presenting to the ED following mTBI, from which prior work has focused on biomarker-based prediction of CT findings (21–24). Here, we extend the objective to address the prediction of P-PCS at 3 months post-injury. P-PCS were assessed using the Parent version of the Post Concussion Symptom Inventory (PCSI-P) at 14 days and 3 months. The objective was to explore the performance of seven brain and inflammatory biomarkers previously described in the t-BIOMAP pediatric population (S100b, NfL, NTproBNP, GFAP, HFABP, IL6 and IL10) (21–24) to identify children with P-PCS after mTBI. The seven biomarkers selected for this study reflect distinct pathophysiological mechanisms relevant to mTBI. S100b and GFAP are established markers of astrocytic injury and have been the most extensively studied in the context of TBI diagnosis. NfL reflects axonal damage and has shown sensitivity to cumulative neuronal injury (25). H-FABP is a marker of vascular injury and has demonstrated predictive value for complete recovery in adult mTBI (18). NT-proBNP has been associated with injury severity in TBI, potentially reflecting cardiovascular stress responses (26). The inflammatory cytokines IL6 and IL10 show central roles in the neuroinflammatory cascade triggered by mTBI (19). IL6 is a pleiotropic pro-inflammatory cytokine rapidly released following brain trauma, contributing to the acute phase response and blood–brain barrier (BBB) disruption, and has been associated with injury severity and early neurological outcomes (20). IL10 is the principal anti-inflammatory cytokine of the post-traumatic response, released by monocytes and macrophages to regulate and limit the pro-inflammatory cascade. While this counter-regulatory response is initially protective, elevated and sustained IL10 levels may reflect the magnitude of the initial neuroinflammatory insult and have been associated with worse functional outcomes in adult TBI (18, 27). None of these proteins have been investigated as a predictor of P-PCS in children, representing a significant gap in the pediatric TBI biomarker literature.

We hypothesize that early biomarker profiles may help differentiate children whose symptoms will persist from those who will recover, thereby offering a clinically useful stratification tool at the time of ED discharge.

## 2 Materials and methods

### 2.1 Study population

Children aged 0 to 16 years presenting with mTBI within 24 h of injury to one of the participating pediatric EDs were included. Three French-speaking hospitals in Switzerland were part of the study (Geneva University Hospitals, Fribourg Hospital HFR, Neuchâtel Hospital (RHNE)). Institutional review board approval was obtained and the study was conducted in accordance with Good Clinical Practice guidelines and the Declaration of Helsinki. The study was registered at www.clinicaltrials.gov: NCT06233851. Informed consent was obtained from parents or legal guardians, as well as from children aged 14 years or older.

mTBI was defined as any of the following: Head trauma with (1) a GCS score of 14; or (2) a GCS score of 15 with at least one of the following symptoms: loss of consciousness (LOC), post-traumatic amnesia (PTA), persistent headaches, irritability, three or more episodes of vomiting, confusion, dizziness or vertigo, post-traumatic seizure, or transient neurological abnormalities; or (3) evidence of a basilar skull fracture; or (4) a severe mechanism of injury, such as a traffic accident or a fall from a height greater than 0.9 m in children under 2 years old, or over 1.5 m in children aged 2 years or older (12). Exclusion criteria included: participation in another clinical study involving pharmacological treatment, recent alcohol consumption or psychoactive substance use, a TBI within the preceding month, seizures within the last month, a diagnosis of Down syndrome, acute encephalopathy, encephalitis, meningitis, or refusal to provide consent.

### 2.2 Intervention and data collection

After informed consent was obtained, a blood sample was drawn shortly after the head trauma (within 24 h), for acute analysis. All participating hospitals followed the same protocol for blood collection and processing. Serum samples were prepared using a standardized centrifugation protocol (2000-3000 g during 10 min at room temperature) and stored as frozen serum aliquots. The study did not interfere with any medical decision-making. Clinical data collected from medical records included: sex, age, GCS score, mechanism of injury, history of coagulation disorders with medication intake prior to trauma, TBI-associated symptoms, presence of simple skull fractures, extracranial injuries (ECIs) such as other body fractures or organs injuries, time between trauma and blood sampling, physician decision regarding patient management (observation without CT scan or undergoing CT scan), duration of ED observation for symptom monitoring, need for neurosurgery and intubation, and results of imaging. When available, C-reactive protein (CRP) levels were recorded as a marker of inflammation and infection at the time of blood sampling. CRP values were used to screen for the presence of an intercurrent infectious or inflammatory condition that could confound biomarker interpretation. Children with markedly elevated CRP suggesting an active systemic inflammatory process were flagged; however, given the small sample size and the exploratory nature of the study, CRP was not formally incorporated as a covariate in the logistic regression models. Study data were collected and managed using REDCap electronic data capture tools hosted at Hôpitaux Universitaires de Genève (HUG) (28, 29).

### 2.3 Assessment of post-concussion symptoms

PCS were assessed using the PCSI-P, as originally developed and validated by Gioia and colleagues (30). The PCSI-P questionnaire was administered electronically via REDCap to parents (or caregiver) at two time points: 14 days and 3 months after the ED visit. The PCSI-P comprises 20 symptoms organized across four subscales reflecting the known factor structure of post-concussive complaints: physical, cognitive, emotional, and fatigue/sleep-related symptoms. The PCSI has demonstrated strong psychometric properties, including high internal consistency (*α* = 0.8–0.9), good test–retest reliability, and acceptable convergent validity with other concussion symptom measures. For each item, parents rated both pre-injury (retrospective baseline) and current symptom severity using a 7-point Guttman scale (0 = no problem, 3 = moderate problem, 6 = severe problem). The difference between current and baseline ratings (delta score) reflects injury-attributable symptom burden. Children were classified as symptomatic if at least three symptoms showed an increase in severity of at least one point compared to the pre-injury baseline (≥3 items with a delta score ≥1). This threshold is in line with the definition used in the Take C. A. Re study (31).

### 2.4 Primary outcome

The primary clinical outcome was persistent PCS (P-PCS), defined as meeting the symptomatic classification threshold at both 14 days and 3 months post-injury. Children who were symptomatic at 14 days but no longer at 3 months were classified as recovered. The primary analysis compared these two subgroups (children with P-PCS versus those who recovered by 3 months). Restricting the analysis to children who first reported PCS at 14 days post-injury, aligns with the established definition of P-PCS, which requires symptom persistence across multiple timepoints rather than *de novo* symptom emergence at 3 months (6, 31). This design choice enriches the comparison of clinically relevant cases, those children who will need to be managed for persistent symptoms. Children who first reported symptoms at 3 months without prior symptoms reported at 14 days could not be classified as having persistent symptoms, as the temporal continuity required for P-PCS diagnosis could not be confirmed.

### 2.5 Blood-based biomarker analysis

Serum samples were obtained by centrifugation and stored locally at −80 °C, before being shipped in batches to the University of Geneva for analysis. Concentrations of IL6, IL10, NfL, NTproBNP, GFAP, S100b, and HFABP were measured using enzyme-linked immunoassay (ELISA): R-plex Human NfL (F217X), NTproBNP (F214I), GFAP (F211M), S100b (F212E), and FABP3/HFABP (F214T) antibody sets; and V-plex Proinflammatory Panel 1 (Human) with IL6 and IL10 antibodies sets, on the MesoQuickPlex SQ120 reader (Meso Scale Diagnostics, Rockville, MD, USA). Lower limit of detection (LLoD) was, respectively, 5.5 pg./mL with a calibration range of 12.21–50,000 pg./mL for NfL; 0.30 pg./mL and 0.12–500 pg./mL for NTproBNP; 63 pg./mL and 122–500,000 pg./mL for GFAP; 1.6 pg./mL and 1.22–5,000 pg./mL for S100b; 90 pg./mL and 24.41–100,000 pg./mL for HFABP; 0.06 pg./mL and 0.06–488 pg./mL for IL6; and finally 0.04 pg./mL and 0.04–233 pg./mL for IL10. The lower limit of quantification (LLoQ) was determined as the minimum concentration with a coefficient of variation (CV) under 20% and a recovery rate between 80 and 120%. Duplicate control serum samples were analyzed on each plate, ensuring that intra- and inter-plate coefficients of variation (CVs) remain below 20%. All kits were utilized following the manufacturers’ guidelines.

### 2.6 Statistical analysis

Statistical analysis was performed using R (http://www.rproject.org, version 4.5.1) in RStudio (http://www.rstudio.com, version 2026.01.0). Biomarker concentrations were normalized using their medians as correction factors. Patients were first dichotomized into two groups: (1) patients with PCS at 14 days post injury (PCS + 14d); and (2) patients without PCS at 14 days post injury (PCS-). Among patients with PCS + at 14 days post injury, a second classification was performed based on 3-month outcomes: persistent PCS (P-PCS) or recovery. Given non-normal distribution of biomarker concentrations (Kolmogorov–Smirnov test, *p* < 0.05), group comparisons were performed using the Mann–Whitney U test and Benjamini-Hochberg correction for multiple comparisons is only shown in tables for transparency. Chi-squared test and Fisher’s exact test were used for statistical analyses of clinical data. Statistical significance was inferred at *p* < 0.05. The levels of biomarkers were visualized using box- and dot-plots with a log10 Y-scale. For logistic regression analysis, biomarker concentrations were log-transformed and standardized (mean = 0, standard deviation = 1). Separate univariable logistic regression models were fitted for each biomarker individually to avoid overfitting. Odds ratios (OR) were used to represent the change in odds of P-PCS per one SD increase in the log-transformed biomarker concentration and were presented with 95% confidence intervals (CI). Biomarkers’ ability for classifying patients according to their group was evaluated using receiver operating characteristic (ROC) curves and optimal Youden index, with the pROC package in R. 95% CI for the area under the curve (AUC) were computed using bootstrap resampling to account for small sample sizes. Two thresholds of clinical interest were pre-specified on the ROC curve: (1) 100% specificity (SP), corresponding to a 100% positive predictive value (PPV), enabling a rule-in strategy to identify with certainty children who will develop P-PCS and should be prioritized for neuropsychological follow-up; and (2) 100% sensitivity (SE), corresponding to a 100% negative predictive value (NPV), enabling a rule-out strategy to safely exclude children at low risk of P-PCS, avoiding unnecessary referrals. These two strategies reflect complementary clinical needs at ED discharge in the context of pediatric mTBI. Given the exploratory nature of the study, analyses were not adjusted for multiple comparisons, increasing the probability of false-positive findings. Due to the limited sample size, findings should be interpreted as hypothesis-generating potential model instability. These analyses should not be used to draw definitive conclusions about the predictive value of individual biomarkers but rather to explore potential of biological markers. Only patients with complete data for all tested biomarkers and questionnaire responses were included in the analysis.

## 3 Results

**Figure 1:**
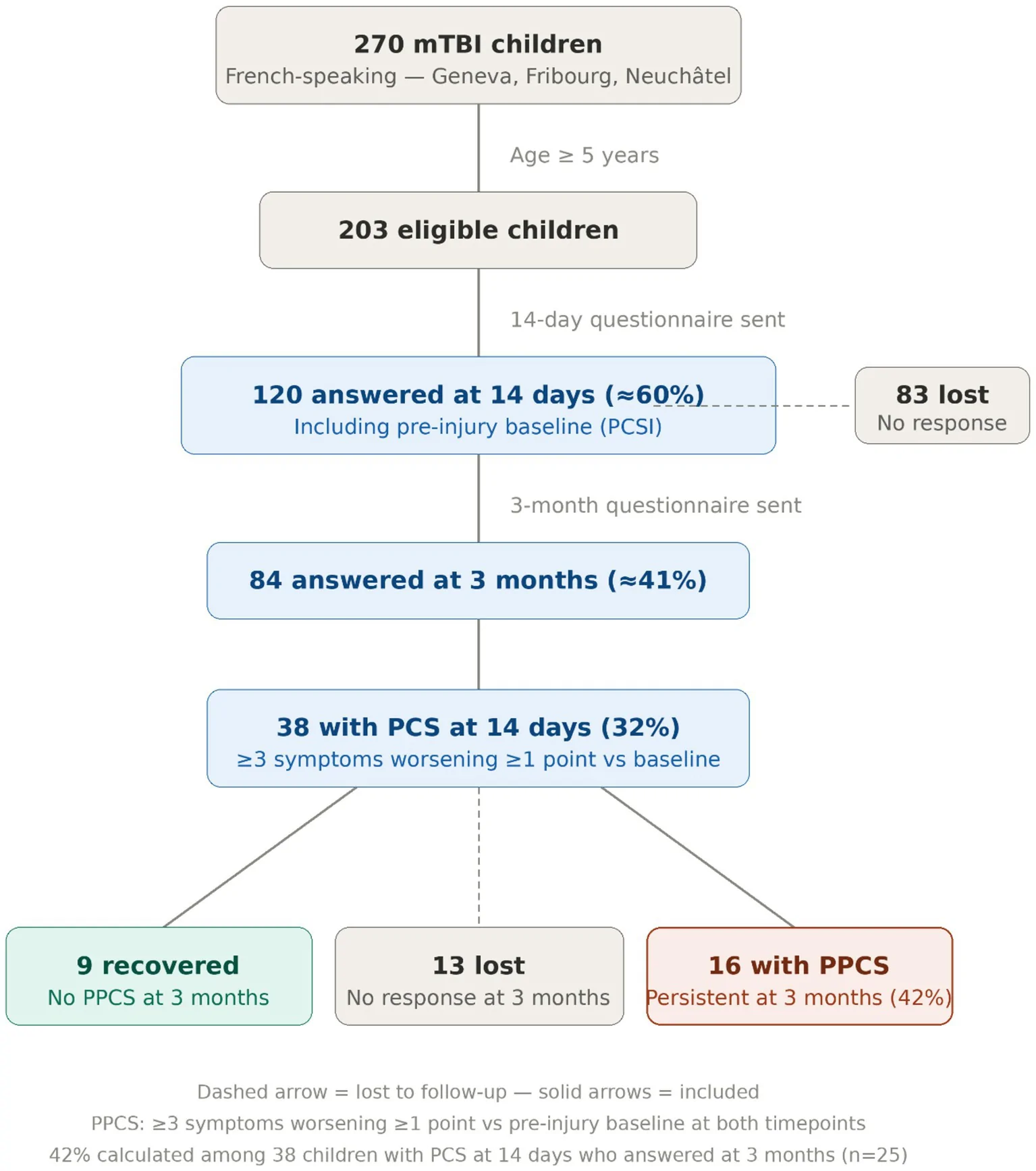
Flowchart of patient selection and follow-up in the t-BIOMAP study. mTBI, mild traumatic brain injury; PCSI, Post-Concussion Symptom Inventory; PPCS, persistent post-concussion symptoms (defined as ≥3 symptoms worsening by ≥1 point above pre-injury baseline at both 14 days and 3 months post-injury).

Among participants enrolled in the t-BIOMAP study, 270 children were French-speaking, of whom 203 were aged 5 years or older and received the questionnaires. Out of these, 120 families completed the 14-day questionnaire, which included the baseline measurement (60% of responders). Subsequently, 84 families answered the 3-month questionnaire (41% of responders). The patient’s selection flowchart is presented in Figure 1.

At 14 days post-injury, 82 (68%) children did not report PCS (PCS-) and 38 (32%) children reported PCS (PCS + 14d). Clinical parameters and biomarkers expression in these two compared groups are summarized in Table 1. No significant differences were observed between groups regarding age, sex, severity of injury (GCS), presence or absence of symptoms criteria for the diagnosis of mTBI such as LOC, PTA, headaches, etc. Likewise, no significant differences in biomarker concentrations were observed between the two groups (Table 1).

**Table 1:** Clinical parameters and biomarker expression in mTBI children with or without PCS at 14 days.

Variables	PCS- (*N* = 82)	PCS+ 14d (*N* = 38)	*p*-value	Adjusted *p*-value (BH) for biomarkers
Inclusion criteria				
Severity of injury, n (%)				
GCS14	8 (9.8%)	2 (5.3%)	0.636	
GCS15	74 (90.2%)	36 (94.7%)		
Sign of basilar skull fracture, n (%)	3 (3.7%)	0 (0%)	0.572	
Severe mechanism of injury*, n (%)	36 (43.9%)	17 (44.7%)	1	
Symptoms at admission (within GCS 15), n (%)				
Loss of consciousness	13 (15.9%)	10 (26.3%)	0.324	
Post-traumatic amnesia	35 (42.7%)	14 (36.8%)	0.491	
Persistent headaches	32 (39.0%)	16 (42.1%)	0.871	
More than three episodes of vomiting	11 (13.4%)	5 (13.2%)	1	
Vertigo	9 (11.0%)	6 (15.8%)	0.684	
Confusion	14 (17.1%)	6 (15.8%)	1	
Convulsion	1 (1.2%)	0 (0%)	1	
Other clinical variables				
Age (yo)				
Mean (SD)	10.1 (3.48)	11.4 (2.84)	0.052	
Median [Min, Max]	9.75 [5.00, 15.9]	11.8 [5.10, 15.5]		
Sex				
Boys	50 (61.0%)	17 (44.7%)	0.142	
Girls	32 (39.0%)	21 (55.3%)		
Extracranial injury (ECI), n (%)	10 (12.2%)	7 (18.4%)	0.53	
CT result				
mTBI in Obs. without CT	70 (85.4%)	29 (76.3%)	0.303	
mTBI with CT-	11 (13.4%)	7 (18.4%)		
mTBI with CT+	1 (1.2%)	2 (5.3%)		
Observation time				
<=8 h	60 (73.2%)	29 (76.3%)	0.731	
>8 h-12 h	5 (6.1%)	3 (7.9%)		
>12 h-18 h	9 (11.0%)	4 (10.5%)		
>18 h-24 h	4 (4.9%)	0 (0%)		
>24 h	4 (4.9%)	2 (5.3%)		
Biomarker expression				
S100b (pg/ml)				
Mean (SD)	57.2 (78.9)	38.1 (39.2)	0.057	0,274
Median [Min, Max]	42.6 [1.17, 672]	28.5 [2.29, 223]		
GFAP (pg/ml)				
Mean (SD)	469 (717)	540 (701)	0.899	0.956
Median [Min, Max]	183 [2.61, 4,620]	186 [4.02, 2,680]		
NTproBNP (pg/ml)				
Mean (SD)	84.2 (70.5)	89.7 (82.6)	0.955	0.956
Median [Min, Max]	61.8 [3.00, 287]	57.8 [6.89, 385]		
HFABP (pg/ml)				
Mean (SD)	3,320 (2790)	2,860 (2070)	0.535	0.956
Median [Min, Max]	2,320 [958, 15,000]	2,350 [237, 9,650]		
NfL (pg/ml)				
Mean (SD)	19.2 (15.3)	19.6 (12.0)	0.456	0.956
Median [Min, Max]	16.0 [0.119, 116]	17.4 [3.70, 69.8]		
IL6 (pg/ml)				
Mean (SD)	2.02 (3.07)	1.73 (2.11)	0.636	0.956
Median [Min, Max]	1.05 [0.218, 16.9]	0.877 [0.0771, 10.1]		
IL10 (pg/ml)				
Mean (SD)	0.885 (2.05)	0.573 (0.992)	0.068	0,274
Median [Min, Max]	0.387 [0.0769, 16.4]	0.266 [0.0634, 5.76]		

*Severe mechanism of injury: such as a traffic accident or a fall from a height greater than 0.9 m in children under 2 years old, or over 1.5 m in children 2 years or older. BH, Adjusted *p*-value with Benjamini-Hochberg correction.

Of the 84 answers obtained at 3 months, 55 children did not report PCS (65%) and 29 reported PCS (35%). However, for the purpose of this study and to increase the likelihood that the symptoms were related to the head injury, only children reporting PCS at both timepoints were classified as having persistent PCS (P-PCS). The analysis was therefore restricted to the 38 children who had already reported PCS at 14 days (PCS + 14d).

Within this subgroup of 38 children, 16 (42%) still reported PCS at 3 months and were defined as the P-PCS group, whereas 9 (24%) had spontaneously recovered at 3 months, and 13 (34%) did not answer the 3-month questionnaire and were considered lost to follow-up. Children who reported PCS at 3 months but had either not responded or did not report PCS at the 14-day questionnaire were not included in the analysis, as persistent symptomatology could not be confirmed across both timepoints.

**Figure 2:**
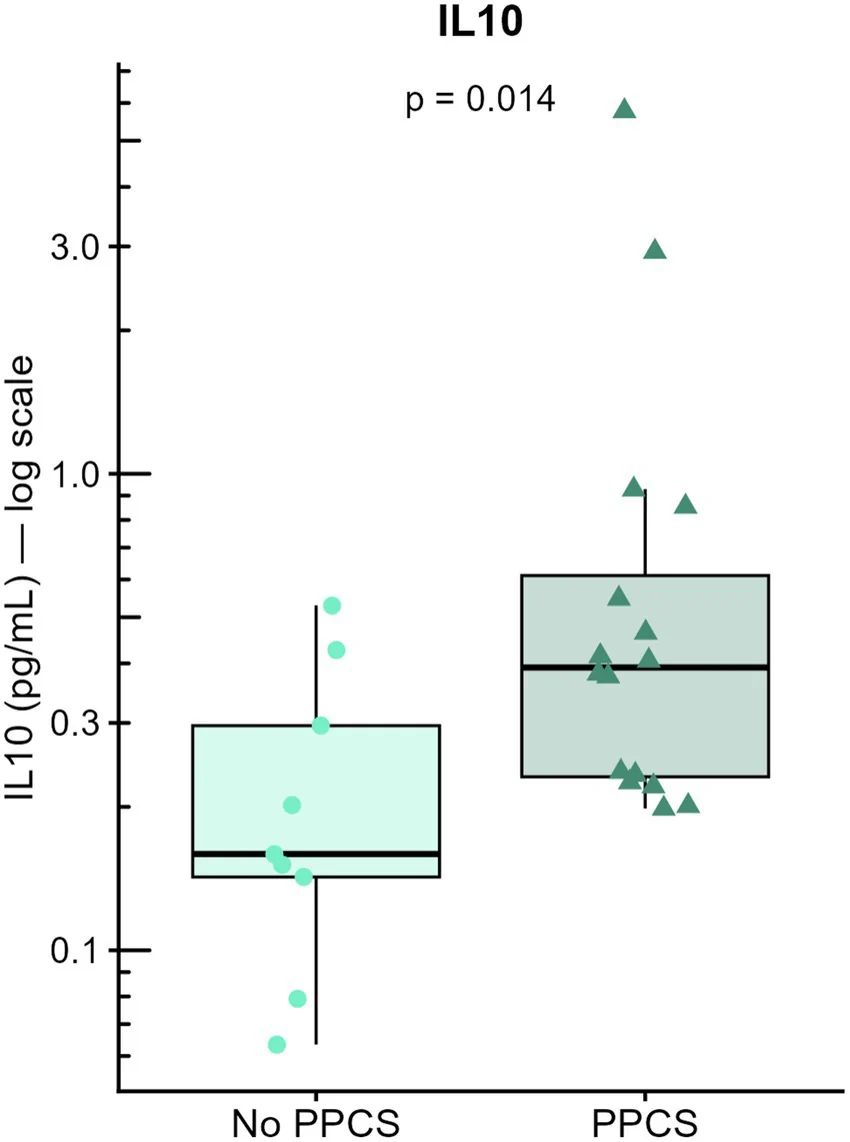
IL10 expression in children with mTBI according to their 3-month post-concussion outcome. Individual values are shown as dots (circles: no PPCS; triangles: PPCS). The box represents the interquartile range, the horizontal line the median. Y-axis is on a log scale. PPCS, persistent post-concussion symptoms (defined as ≥3 PCSI symptoms worsening by ≥1 point above pre-injury baseline at both 14 days and 3 months post-injury). *p*-value from Wilcoxon rank-sum test.

**Figure 3:**
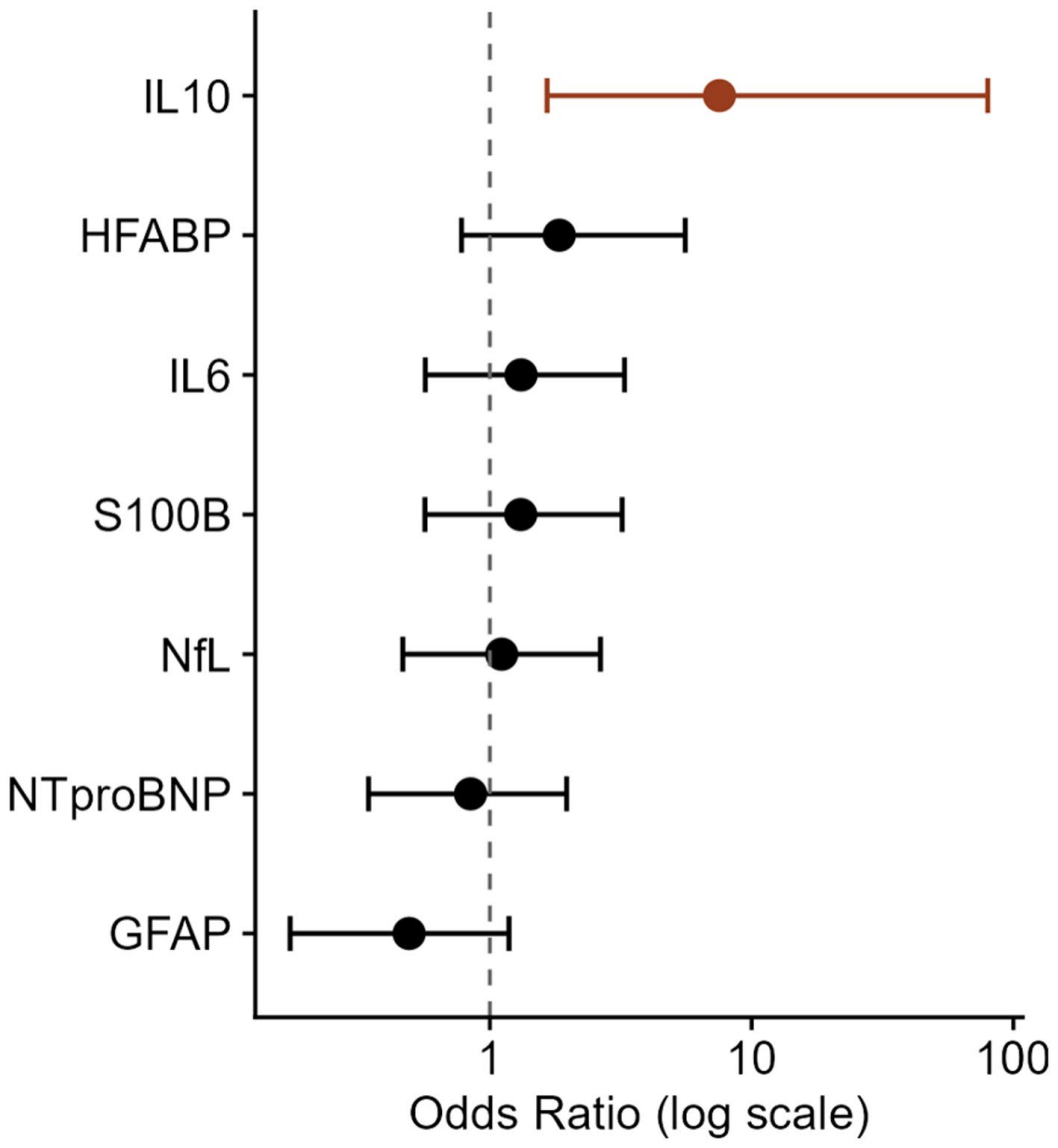
Forest plot of odds ratios (OR) for the association between early blood biomarker expressions and persistent post-concussion symptoms (PPCS) at 3 months. OR per 1 standard deviation increase in log-transformed biomarker concentration. Error bars represent 95% confidence intervals. The dashed line indicates OR = 1. The red point indicates the only biomarker reaching statistical significance (*p* < 0.05). OR, odds ratio; PPCS, persistent post-concussion symptoms.

Clinical parameters and biomarker expressions in the two comparison groups are presented in Table 2. Among the clinical parameters assessed, the presence of more than three vomiting episodes was the only variable that significantly differed between groups (*p* = 0.033), being more frequent in children who recovered by 3 months. No other clinical parameter reached statistical significance. Of the biomarkers analyzed, only IL10 differed significantly between groups. IL10 concentration was significantly higher in children with P-PCS than in those who recovered (*p* = 0.014). Boxplot of IL10 expressions in the two compared groups is presented in Figure 2. Boxplots of the other biomarkers are available in Supplementary Figure S1. In logistic regression analysis, IL10 was the only biomarker significantly associated with P-PCS at 3 months (OR = 7.54 per 1 SD increase in log-transformed concentration, 95% CI [1.65–79.8], *p* = 0.036). No significant association was observed for the other six biomarkers tested. OR representing the association with all biomarker’s expressions and presence of P-PCS are presented in Figure 3.

**Table 2:** Clinical parameters and biomarker expression in mTBI children with or without PCS at 3 months.

	No P-PCS (*N* = 9)	P-PCS (*N* = 16)	*p*-value	Adjusted *p*-value (BH) for Biomarkers
Inclusion criteria				
Severity of injury, n (%)				
GCS14	1 (11.1%)	0 (0%)	0.766	
GCS15	8 (88.9%)	16 (100%)		
Sign of basilar skull fracture, n (%)	0 (0%)	0 (0%)		
Severe mechanism of injury*, n (%)	3 (33.3%)	6 (37.5%)	1	
Symptoms at admission (within GCS 15), n (%)				
Loss of consciousness	2 (22.2%)	5 (31.3%)	1	
Post-traumatic amnesia	3 (33.3%)	5 (31.3%)	1	
Persistent headaches	3 (33.3%)	6 (37.5%)	1	
More than three episodes of vomiting	3 (33.3%)	0 (0%)	**0.033**	
Vertigo	1 (11.1%)	2 (12.5%)	1	
Confusion	0 (0%)	3 (18.8%)	0.578	
Convulsion	0 (0%)	0 (0%)		
Other clinical variables				
Age (yo)				
Mean (SD)	12.1 (1.84)	11.0 (3.22)	0.552	
Median [Min, Max]	12.1 [9.30, 15.5]	11.6 [5.10, 14.7]		
Sex				
Boys	4 (44.4%)	6 (37.5%)	1	
Girls	5 (55.6%)	10 (62.5%)		
Extracranial injury (ECI), n (%)	0 (0%)	3 (18.8%)	0.457	
CT result				
mTBI in Obs. without CT	6 (66.7%)	13 (81.3%)	0.373	
mTBI with CT-	2 (22.2%)	3 (18.8%)		
mTBI with CT+	1 (11.1%)	0 (0%)		
Observation time				
<=8 h	6 (66.7%)	12 (75.0%)	NA	
>8 h-12 h	1 (11.1%)	1 (6.3%)		
>12 h-18 h	1 (11.1%)	3 (18.8%)		
>18 h-24 h	0 (0%)	0 (0%)		
>24 h	1 (11.1%)	0 (0%)		
Biomarker expression				
S100b (pg/ml)				
Mean (SD)	32.8 (22.5)	50.7 (54.6)	0.559	0.655
Median [Min, Max]	33.1 [4.28, 69.3]	32.9 [4.84, 223]		
GFAP (pg/ml)				
Mean (SD)	870 (1050)	339 (448)	0.152	0.499
Median [Min, Max]	238 [21.6, 2,680]	96.2 [5.84, 1,540]		
NTproBNP (pg/ml)				
Mean (SD)	111 (115)	83.3 (73.7)	0.533	0.655
Median [Min, Max]	57.9 [6.89, 385]	53.6 [15.7, 245]		
HFABP (pg/ml)				
Mean (SD)	1880 (875)	3,510 (2750)	0.187	0.499
Median [Min, Max]	1830 [237, 3,100]	2,550 [250, 9,650]		
NfL (pg/ml)				
Mean (SD)	20.0 (8.85)	23.0 (15.1)	0.677	0,677
Median [Min, Max]	16.7 [5.75, 33.5]	19.1 [3.70, 69.8]		
IL6 (pg/ml)				
Mean (SD)	1.47 (1.88)	1.97 (2.65)	0.452	0.655
Median [Min, Max]	0.613 [0.125, 5.73]	1.16 [0.0771, 10.1]		
IL10 (pg/ml)				
Mean (SD)	0.228 (0.159)	0.898 (1.46)	**0.014**	0.111
Median [Min, Max]	0.159 [0.0634, 0.529]	0.392 [0.198, 5.76]		

*Severe mechanism of injury: such as a traffic accident or a fall from a height greater than 0.9 m in children under 2 years old, or over 1.5 m in children 2 years or older. BH, Adjusted *p*-value with Benjamini-Hochberg correction. Bold values are significant *p*-values (*p*-value < 0.05).

**Figure 4:**
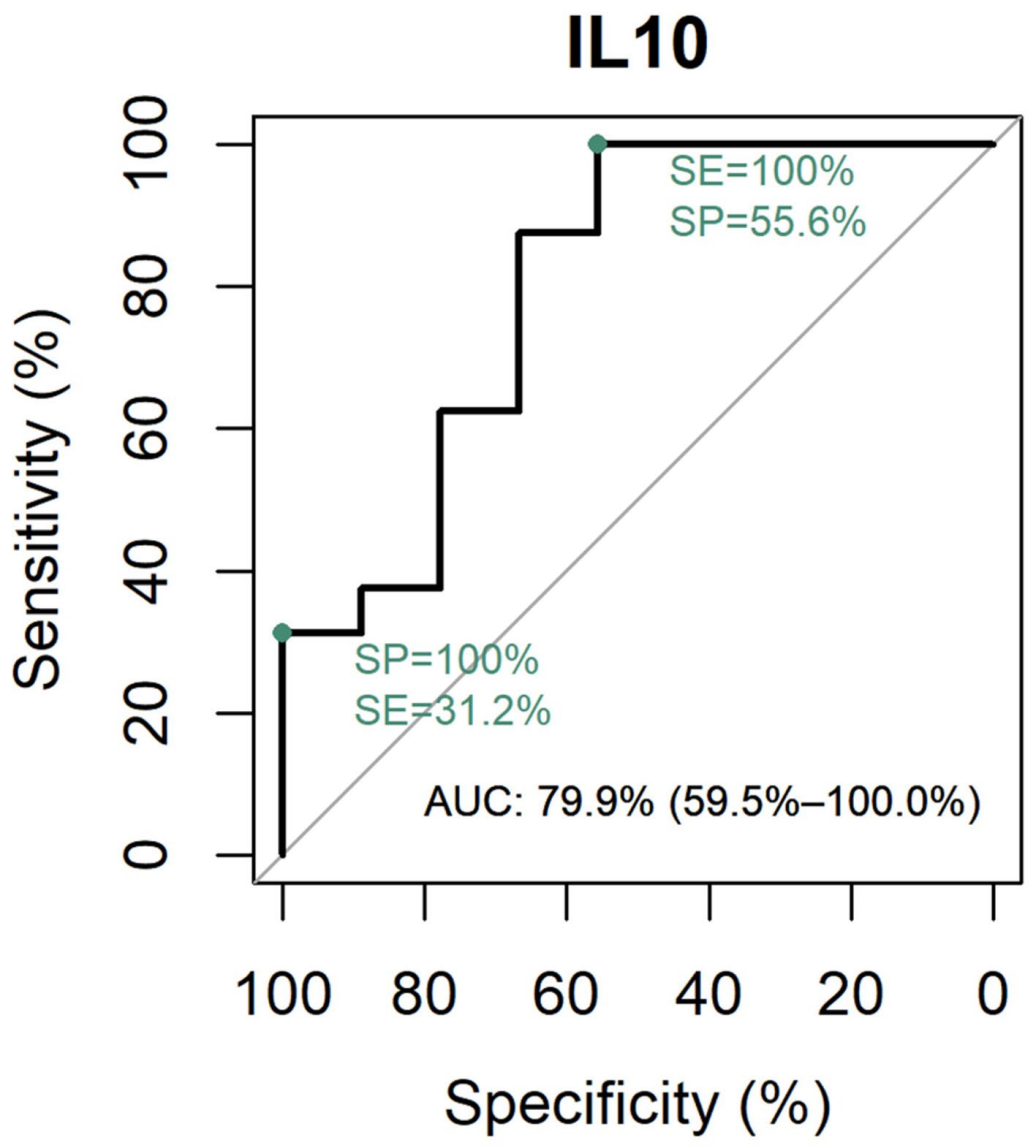
IL10 receiver operating characteristic (ROC) curve for predicting persistent post-concussion symptoms (P-PCS) at 3 months. The green circles indicate the operating point at 100% specificity (SP; positive predictive value = 100%, sensitivity (SE) = 31.3%) and at 100% sensitivity (SE; negative predictive value = 100%, specificity (SP) = 55.6%). AUC, area under the curve.

The performance of IL10 to predict P-PCS at 3 months is illustrated with the ROC curve in Figure 4. The AUC for IL10 was 79.9% (95% CI: 59.5–100%). At the optimal Youden index threshold, IL10 achieved 100% SE and 55.6% SP. Conversely, at 100% SP, IL10 achieved 31.3% SE. These two operating points reflect complementary clinical risk stratification strategies. In this exploratory cohort, a potential rule-out approach (100% negative predictive value [NPV], 55.6% SP) could safely discharge children at lower risk of P-PCS, and a rule-in approach (100% positive predictive value [PPV], 31.3% SE) could identify children at higher risk of P-PCS, requiring neuropsychological follow-up. These estimates carry wide 95% CI, reflecting substantial statistical uncertainty.

Based on this exploratory analysis, there are potential directions for clinical practice. At the rule-out threshold, half of children who will not develop P-PCS could be safely discharged, while all children at higher risk of P-PCS could be captured. Conversely, at the rule-in threshold, one third of children who will develop P-PCS could be identified without false positives.

## 4 Discussion

This study investigated the association between early blood biomarker levels and the development of P-PCS at 3 months in children presenting to the ED following mTBI. Our main finding is that IL10 concentrations, measured within 24 h of injury, were significantly elevated in children who reported PCS at both 14 days and 3 months compared to those who had recovered by 3 months, yielding an AUC of 79.9% (95% CI: 59.5–100%). Two clinically relevant thresholds were identified on the ROC curve: at 100% SP, IL10 achieved 31.3% SE, enabling a rule-in strategy to identify with certainty children at risk of developing P-PCS; conversely, at 100% SE, IL10 achieved 55.6% SP, supporting a rule-out strategy to safely exclude children without risk of P-PCS. To our knowledge, this is the first study to demonstrate a potential predictive role for IL10 in the outcome of pediatric mTBI, offering two complementary risk-stratification strategies that could potentially be applied at ED discharge Given the exploratory nature of the analyses and small sample size, uncertainty around the observed association should be considered as a preliminary signal requiring replication, rather than a definitive estimate of effect size.

After ruling out an intracranial injury requiring acute management in the context of pediatric mTBI, the primary challenge at ED discharge is not simply to identify children who will recover spontaneously, since most do so, but rather to selectively identify those at risk of developing persistent symptoms who may benefit from structured neuropsychological follow-up.

In this study, IL10 yielded an AUC of 79.9% (95% CI: 59.5–100%) in predicting P-PCS at 3 months. Two clinically relevant thresholds were identified, each corresponding to a distinct risk-stratification strategy applicable at ED discharge. The first was a rule-in approach, which may be particularly relevant in healthcare settings where structured neuropsychological follow-up after pediatric mTBI is not routinely organized, and specialist resources remain limited. In such a context, this threshold could serve as a reliable trigger for referrals, ensuring that children referred for specialized follow-up are highly likely to require it. The second strategy was a rule-out approach that could be used to safely reassure families and discharge children without follow-up. This strategy is ethically compelling as it minimizes the likelihood of missing children who may subsequently require specialist care.

These two strategies address complementary clinical needs. A rule-in strategy may be preferable in resource-constrained systems where avoiding unnecessary referrals is essential, whereas a rule-out strategy may be favored when the priority is to ensure that no child at risk of future P-PCS is lost to follow-up.

Among the candidate biomarkers with potential relevance to post-injury neuroinflammatory processes, IL10 has attracted growing interest. IL10 is a pleiotropic anti-inflammatory cytokine that plays a modulatory role in the central nervous system response to injury and has been implicated in the regulation of neuroinflammation following TBI (19). The significant association observed in our cohort between elevated early IL10 and persistent symptoms at 3 months is consistent with findings previously reported in adults. Elevated early IL10 levels have been associated with worse functional outcomes and increased mortality after TBI (32), consistent with the hypothesis that higher IL10 concentrations may reflect the magnitude of the initial neuroinflammatory response and may serve as a marker of injury severity. Lagerstedt et al. demonstrated that IL10, measured within 24 h of TBI, showed the highest specificity (50%) for predicting unfavorable outcome (GOSE ≤ 4) at 6 months among patients with all TBI severities, when sensitivity was fixed at 95–100% (18). That same study also evaluated HFABP, one of the seven biomarkers in our panel, and found it to be the strongest predictor of complete recovery specifically in mTBI patients. The fact that HFABP did not reach significance in our pediatric cohort may reflect differences in outcome measure, patient age, injury severity distribution, or the small sample size. Nevertheless, the convergence of IL10 as a relevant signal in both the adult literature and our pediatric data strengthens the hypothesis that the early anti-inflammatory cytokine response may represent a biologically meaningful determinant of recovery trajectory after mTBI, across age groups and outcome measures (19, 20). Moreover, a recent study in pediatric patients with head trauma further supports the role of neuroinflammatory and vasoactive mediators in TBI outcomes (33), consistent with our finding that the anti-inflammatory cytokine IL10 was significantly elevated in children who developed P-PCS.

None of the other biomarkers evaluated in our study, S100b, GFAP, NfL, NTproBNP, HFABP, and IL6, reached statistical significance in predicting P-PCS at 3 months. Interestingly, GFAP demonstrated a non-significant trend toward lower concentrations in children who developed P-PCS (OR = 0.49), which is consistent with previous reports suggesting that higher acute GFAP levels may reflect a more robust glial response associated with better tissue repair after mTBI (34, 35).

Consistent with the existing literature, clinical parameters collected at the time of ED presentation, including age, sex, GCS score, LOC, PTA, persistent headaches, or CT findings, did not significantly differentiate children who later developed P-PCS from those who recovered. This finding underscores the well-recognized clinical challenge of identifying children at risk for prolonged recovery using convention clinical assessment alone. In our study, the observed association between repetitive vomiting at presentation and subsequent recovery should be interpreted with caution. Vomiting is a non-specific acute symptom that may primarily reflect the severity of the immediate vestibular response rather than the neuroinflammatory processes underlying persistent symptoms and translating into persistent neurofunctional impairment.

These findings must be interpreted considering both the strengths and limitations of the present study. This study has several noteworthy strengths. It is conducted within the framework of the multicenter t-BIOMAP study, ensuring prospective, standardized data collection across three French-speaking Swiss centers. The use of a validated, PCSI-based binary classifier to define symptomatic status provides a psychometrically valid outcome measure (30, 31). The response rate at 14 days was satisfactory at approximately 60%, reflecting good parental engagement in the acute recovery phase.

However, several limitations must be acknowledged. The most important is the small sample size available for the primary comparison, as only 25 children symptomatic at 14 days (9 recovered at 3 months and 16 with P-PCS) were included in the analysis. This resulted in a wide 95% CI for the OR of IL10, which limits the precision of the estimate, carry substantial uncertainty and precludes definitive conclusions due to the risk of overfitting. The finding should therefore be considered hypothesis-generating, requiring replication in larger prospective cohorts, where adjustment for multiple comparisons can be performed. A comprehensive neuropsychological evaluation would have provided a more complete characterization of P-PCS; however, this was not feasible within the scope and design of the present study, which was conducted in an ED setting with the constraint of a secondary outcome endpoint. The attrition between 14 days and 3 months (a response rate of approximately 41% at 3 months) represents a meaningful source of potential bias, as families who respond at both time points may differ systematically from those lost to follow-up. Systematic differences between responders and non-responders cannot be excluded. A formal comparison of baseline characteristics between children included in the final analysis and those lost to follow-up at 3 months was not performed, which limits our ability to assess the risk of attrition bias. The restriction of the primary analysis to children symptomatic at 14 days, was justified by P-PCS definition requiring temporal continuity of symptoms. While this design choice reduced the evaluable sample size and may have introduced selection bias, it aimed to enrich the comparison with clinically relevant cases, those one that indeed need to be managed for persistent symptoms. This should be addressed in future studies with dedicated follow-up strategies to minimize participant attrition. This challenge is well recognized in prospective pediatric concussion research and highlights the need for sustained engagement strategies in future studies. A further limitation concerns the use of a single biomarker measurement obtained within the first 24 h after injury. While it may reflect a true absence of predictive association, it is equally possible that the timing of sampling was not optimal for all molecules studied. Biomarkers of structural neuronal injury such as NfL are known to peak at different time points post-injury, and kinetic studies in adults suggest that serial measurements over the first days to weeks may provide greater prognostic value than a single acute measurement (36–38). Moreover, while IL10 appears promising as a component of a predictive model, it is unlikely to be sufficient as a standalone biomarker for clinical risk stratification. Future studies should therefore investigate whether combining IL10 with other clinical or biological variables such as age, sex, symptom severity at presentation, and mechanism of injury, within multivariable prediction models could improve overall prognostic performance. Furthermore, given the small sample size and the exploratory nature of the study, CRP was not formally incorporated as a covariate in the logistic regression model. Elevated IL10 may appear paradoxical given that IL10 is primarily known as an anti-inflammatory cytokine. However, several mechanisms may account for this observation. Further mechanistic studies are needed to clarify the biological pathway linking elevated early IL10 to persistent post-concussive symptomatology.

Finally, our cohort was restricted to children aged 5 years and older, as validated symptom assessment tools for younger children remain limited. This represents an important gap in the field. Young children, particularly those under five, may be particularly vulnerable to undetected PCS and its neurodevelopmental consequences, yet symptom identification in this age group remains challenging. The development of age-adapted assessment tools for early childhood brain injury, such as those proposed by Beauchamp and colleagues represents a promising direction for addressing this gap (39).

## 5 Conclusion

This study provides evidence that IL10, measured acutely within 24 h after pediatric mTBI may serve as a candidate blood biomarker for identifying children at risk of developing P-PCS at 3 months. These results should be considered strictly hypothesis-generating and require replication in larger, adequately powered, independent prospective cohorts before IL10 can be considered as a candidate tool to be incorporated into multivariable models combining clinical and biological factors, for clinical risk stratification in pediatric mTBI. However, this finding is consistent with the recognized predictive role of IL10 in adult TBI outcome and supports the hypothesis that the early neuroinflammatory response is a biologically meaningful determinant of recovery in pediatric mTBI. Given that clinical and neuroimaging parameters obtained at ED presentation remain insufficient to predict long-term outcome, and that structured neuropsychological follow-up after pediatric mTBI is often lacking, the identification of a routinely measurable blood biomarker represents a clinically important step toward improved early risk stratification in a population that accounts for a substantial burden of ED visits. Larger prospective studies incorporating serial biomarker sampling, extended age inclusion, and strategies to minimize loss to follow-up are needed to validate and extend these results. Ultimately, a validated and accessible blood-based risk-stratification tool such as IL10 could substantially improve ED discharge practices for pediatric mTBI by ensuring timely identification and referral of children at greatest risk for persistent symptoms.

## Data Availability

The raw data supporting the conclusions of this article will be made available by the authors, without undue reservation.

## Glossary

AUC: Area Under the Curve
BBB: Blood Brain Barrier
CI: Confidence Interval
CT: Computed Tomography
CV: Coefficient of Variation
ECI: Extracranial Injury
ED: Emergency Department
ELISA: Enzyme-Linked Immunoassay
GCS: Glasgow Coma Scale
GFAP: Glial Fibrillary Acidic Protein
HFABP: Heart Fatty-Acid-Binding Protein
HUG: Hopitaux Universitaires de Genève
IC: Confidence Interval
ICI: Intracranial injury
IL10: Interleukin 10
IL6: Interleukin 6
IQR: Inter Quartile Range
LLoD: Lower Limit of Detection
LLoQ: Lower Limit of Quantification
LOC: Loss of Consciousness
Max: Maximum
Min: Minimum
mTBI: mild Traumatic Brain Injury
NfL: Neurofilament Light chain
NPV: Negative Predictive Value
NTproBNP: N-terminal prohormone of brain natriuretic peptide
OR: Odds Ratio
PECARN: Pediatric Emergency Care Applied Research Network
PED: Pediatric Emergency Department
PCSI: Post-Concussion Symptoms Inventory
PPC: Persistent Post-Concussion Symptoms
PTA: Post Traumatic Amnesia
ROC: Receiver Operating Characteristic
S100b: S100 calcium-binding protein B
SE: Sensitivity
SD: Standard Deviation
SP: Specificity
TBI: Traumatic Brain Injury
