# Supplementary material for: Acute interleukin-10 predicts persistent post-concussion symptoms in children with mild traumatic brain injury: exploratory findings from the t-BIOMAP study
Source: Front Neurol. 2026 Aug 18;17:1893730. doi: 10.3389/fneur.2026.1893730 (PMC13529445; doi:10.3389/fneur.2026.1893730)
Supplement: Supplementary file 1 [file Data_Sheet_1.PDF]

SUPPLEMENTAL

Supplementary Figure S1: Biomarkers expression in children with mTBI according to their 3-month post-concussion outcome (S100b, NTproBNP, GFAP, HFABP, NfL, and IL6).

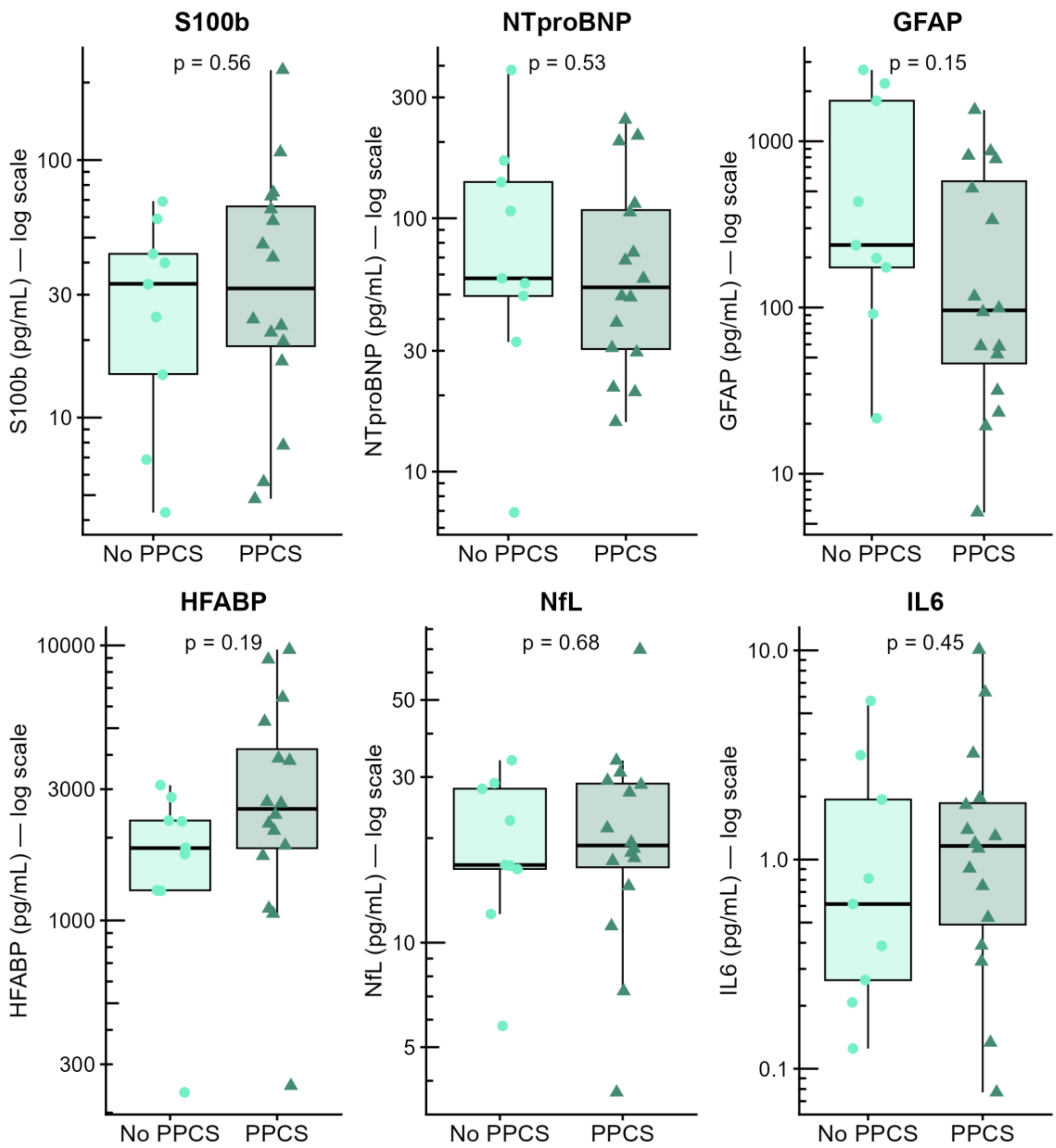

Legend: Individual values are shown as dots (circles: no PPCS; triangles: PPCS). The box represents the interquartile range, the horizontal line the median. Y-axes are on a log scale. PPCS, persistent post-concussion symptoms (defined as ≥3 PCSI symptoms worsening by ≥1 point above pre-injury baseline at both 14 days and 3 months post-injury). p-values from Wilcoxon rank-sum test. No significant differences were observed for any of these biomarkers.
